# 6,6′-Diheptyl-3,3′-bis­[(pyridin-3-yl)ethyn­yl]-5*H*,5′*H*-di­pyrrolo­[1,2-*b*:1′,2′-*g*][2,6]naphthyridine-5,5′-dione

**DOI:** 10.1107/S2414314623005138

**Published:** 2023-06-09

**Authors:** Juan Xiang, Huan Gao, Zhengyu Ma, Xiaohua Cai, Yu-Peng Zhang

**Affiliations:** a College of Chemical Engineering, Guizhou Minzu University, Guiyang,550025,Guizhou, People’s Republic of China; University of Aberdeen, United Kingdom

**Keywords:** crystal structure, cross-conjugated dye, di­pyrrolo, photoluminescence spectroscopy

## Abstract

The complete mol­ecule of the title compound, C_42_H_42_N_4_O_2_, is generated by a crystallographic centre of symmetry. The pendant heptyl chains adopt extended conformations and the dihedral angle between the pyrrole and pyridine rings is 8.18 (15)°. In the crystal, the mol­ecules are arranged in columnar stacks propagating in the [010] direction *via* slipped aromatic π–π stacking inter­actions.

## Abstract 2

The complete mol­ecule of the title compound is generated by a crystallographic centre of symmetry. The pendant heptyl chains adopt extended conformations and the dihedral angle between the pyrrole and pyridine rings is 8.18 (15)°·In the crystal, the mol­ecules are arranged in columnar stacks propagating in the [010] direction *via* slipped aromatic π–π stacking inter­actions.

## Graphical abstract

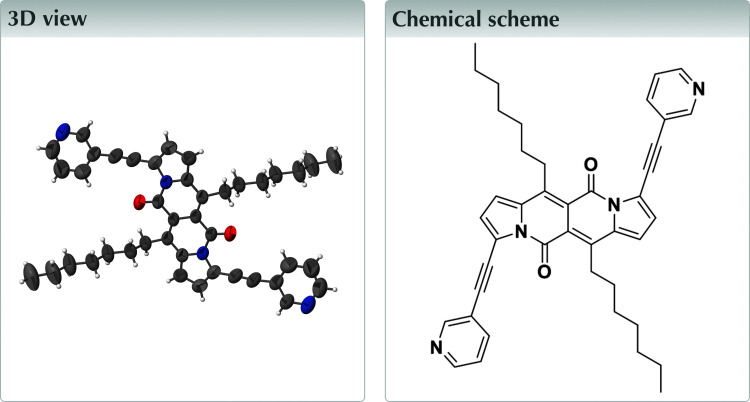

## Structure description

5*H*,11*H*-di­pyrrolo­[1,2-*b*:1′,2′-*g*][2,6]naphthyridine-5,11-dione (C_18_H_16_N_2_O_2_; DPND) is a cross-conjugated dye that has attracted significant attention since it was first reported by Grzybowski *et al.* (2016 ▸). Such a skeleton is composed of electron-rich pyrrole rings and electron-poor carbonyl groups. Several studies have shown that it has inter­esting electrochemical and photophysical properties and it is widely used as a fluorescent dye (Sadowski *et al.*, 2017 ▸; Sadowski, Loebnitz, *et al.*, 2018 ▸; Sadowski, Rode, *et al.*, 2018 ▸). It also has become a potential candidate in singlet fission for enhancing the performance of photo-voltaic devices (Wang *et al.*, 2020 ▸), two-photon absorption materials (Sadowski *et al.*, 2017 ▸) and photodynamic therapy agents (Morgan, Yun, Jamhawi, *et al.*, 2023 ▸). In order to explore the luminescence properties of such mol­ecules in the near infrared region, the strategy of expanding the DPND conjugated system by introducing a pendant pyridine unit was adapted and we synthesized the title compound C_42_H_42_N_4_O_2_, named DPND-3Py, and we now describe its structure and spectroscopic properties.

**Figure 1:**
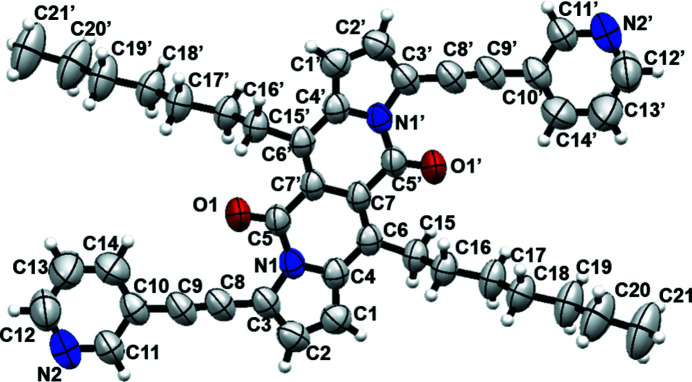
The mol­ecular structure of the title compound with displacement ellipsoids drawn at the 50% probability level. Symmetry code for the primed atoms: 1 − *x*, 2 − *y*, 1 − *z*.

The complete mol­ecule is generated by a crystallographic centre of symmetry (Fig. 1 ▸) and the central chromophore is almost planar (r.m.s. deviation for 16 atoms = 0.028 Å). The pyridine unit is connected to the pyrrole ring of the DPND core by an alkyne bond, which enhances the rigidity of the mol­ecule: the dihedral angle between the N1/C1–C4 and N2/C10–C14 rings is 8.18 (15)°. The pendant heptyl chains adopt extended conformations.

**Figure 2:**
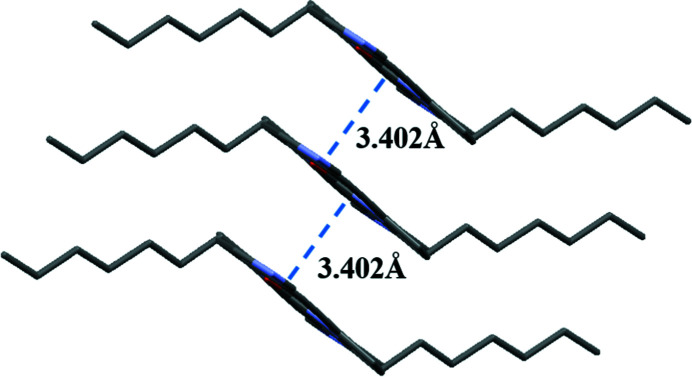
The packing arrangement of the title compound, which shows a slip-stack pattern with a π–π distance of 3.402 Å between the closest planes of these two mol­ecules.

In the extended structure (Fig. 2 ▸), the mol­ecules of the title compound are arranged in [010] columnar stacks *via* slipped aromatic π–π stacking inter­actions with the shortest atom–atom contacts being 3.544 (3) Å for N1⋯C5, 3.613 (3) Å for C4⋯C1 and 3.632 (3) Å for C2⋯C6.

**Figure 3:**
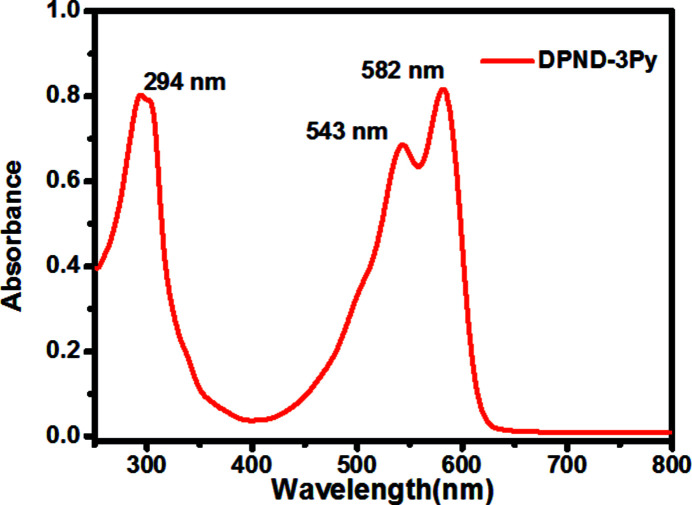
UV–vis absorption spectrum of the title compound.

UV–vis spectra were recorded on a TU-1810DPC spectrometer using di­chloro­methane (DCM) as solvent and a concentration of 2.5 × 10 ^−6^ mol l^−1^. As shown in Fig. 3 ▸, the title compound has three distinct absorption peaks in the range 250 to 800 nm, with a maximum absorption peak of 582 nm. The spectrum features strong absorption in the 500–600 nm region ascribed to an optically allowed *S*_0_ → *S*_1_ transition.

**Figure 4:**
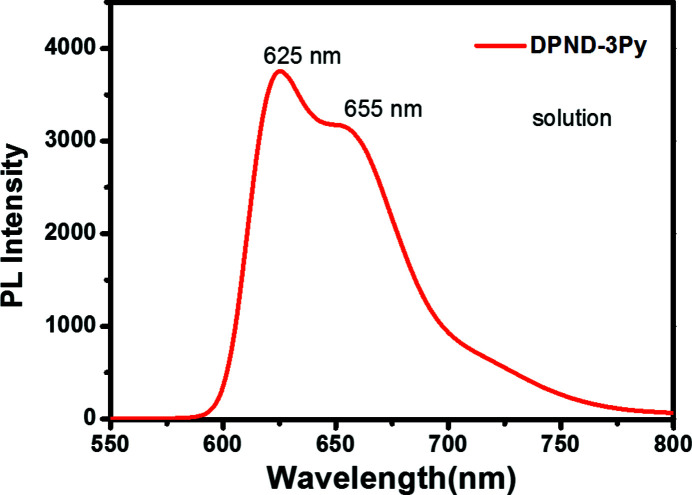
Fluorescence spectrum of the title compound dissolved in DCM.

**Figure 5:**
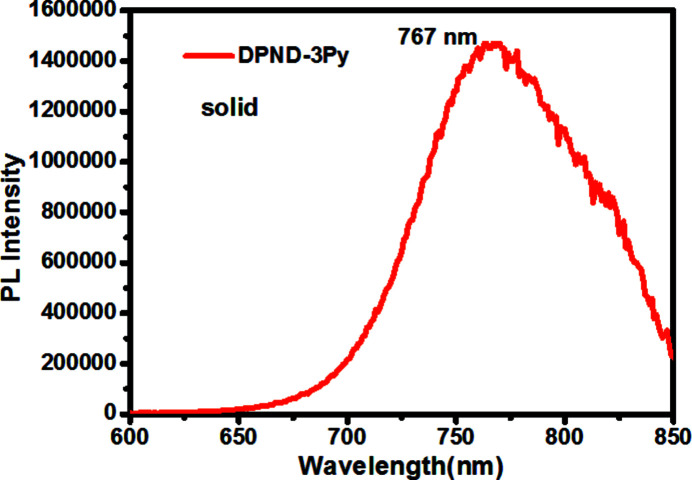
Solid-state emission spectrum of the title compound at an excitation wavelength of 470 nm.

Photoluminescence spectra were recorded on a F-320 spectrometer or HORIB Fluoro­log-3. Figs. 4 ▸ and 5 ▸ show the photoluminescence spectra both in solution (1.0 × 10 ^−5^ mol l^−1^ in di­chloro­methane) and the solid state. The solution spectrum displays two peaks (maximum emission wavelength 625 nm) in the range 550 nm to 800 nm. As shown in Fig. 5 ▸, the solid-state fluorescence spectrum exhibits a strong emission peak at 767 nm, a shift of over 100 nm compared with solution, indicating strong inter­molecular inter­actions.

## Synthesis and crystallization

In a reaction flask containing a magnetic stirring bar was placed: 3,3′-di­bromo-6,6′-diheptyl-5*H*,5′*H*-di­pyrrolo­[1,2-*b*:1′,2′-*g*][2,6]naphthyridine-5,5′-dione (59.04 mg, 0.100 mmol), CuI (1.9 mg, 0.01 mmol), Pd(PPh_3_)_4_ (5.78 mg, 0.005 mmol) and 3-pyridine-acetyl­ene (30.94 mg, 0.300 mmol). The vessel was evacuated and backfilled with argon (three times) and anhydrous, degassed tetra­hydro­furan (THF) was added (3 ml) followed by dry tri­ethyl­amine (56 µl, 0.40 mmol). The vessel was tightly closed and again carefully evacuated (until the mixture started to boil) and backfilled with argon (3 times). The content of the flask was stirred for 20 h at 70°C (above the boiling point), and it was cooled to room temperature. Di­chloro­methane (DCM) was added to dilute the reaction solution, which was washed three times with water and dried over sodium sulfate. The solvent was evaporated and the product was purified using column chromatography (silica, petroleum ether: ethyl acetate = 5:1), and recrystallized from mixed solvents of DCM and methanol to obtain a dark-purple solid (38.5 mg, yield of 35%) (Grzybowski *et al.*, 2016 ▸). Figure S1 in the supporting information shows the ^1^H NMR spectrum of the title compound. The title compound dissolved in methyl­ene chloride and methanol solution grew dark-red crystals suitable for crystallographic studies by slowly volatilizing the solvents.

## Refinement

Crystal data, data collection, and structure refinement details are summarized in Table 1 ▸.

## Supplementary Material

Crystal structure: contains datablock(s) I, global. DOI: 10.1107/S2414314623005138/hb4432sup1.cif

Structure factors: contains datablock(s) I. DOI: 10.1107/S2414314623005138/hb4432Isup3.hkl

Supporting information file. DOI: 10.1107/S2414314623005138/hb4432Isup4.tif

CCDC reference: 2259343

Additional supporting information:  crystallographic information; 3D view; checkCIF report

## Figures and Tables

**Table 1:** Experimental details

Crystal data	
Chemical formula	C_42_H_42_N_4_O_2_
*M*_r_	634.79
Crystal system, space group	Monoclinic, *P*2_1_/*c*
Temperature (K)	300
*a*, *b*, *c* (Å)	12.3973 (4), 4.76620 (15), 31.5382 (10)
β (°)	99.318 (3)
*V* (Å^3^)	1838.94 (10)
*Z*	2
Radiation type	Cu *K*α
μ (mm^−1^)	0.56
Crystal size (mm)	0.24 × 0.06 × 0.04

Data collection	
Diffractometer	XtaLAB Synergy, Dualflex, HyPix
Absorption correction	Multi-scan (*CrysAlis PRO*; Rigaku OD, 2022 ▸)
*T*_min_, *T*_max_	0.288, 1.000
No. of measured, independent and observed [*I* > 2σ(*I*)] reflections	10392, 3568, 2494
*R*_int_	0.035
(sin θ/λ)_max_ (Å^−1^)	0.631

Refinement	
*R*[*F* ^2^ > 2σ(*F* ^2^)], *wR*(*F* ^2^), *S*	0.090, 0.255, 1.05
No. of reflections	3568
No. of parameters	218
H-atom treatment	H-atom parameters constrained
Δρ_max_, Δρ_min_ (e Å^−3^)	0.23, −0.34

Computer programs: *CrysAlis PRO* (Rigaku OD, 2022 ▸), *SHELXT* (Sheldrick, 2015*a* ▸), *SHELXL2014/6* (Sheldrick, 2015*b* ▸) and *Mercury* (Macrae *et al.*, 2020 ▸).

## full crystallographic data

### Crystal data

**Table:**

C_42_H_42_N_4_O_2_	*F*(000) = 676
*M**_r_* = 634.79	*D*_x_ = 1.146 Mg m^−^^3^
Monoclinic, *P*2_1_/*c*	Cu *K*α radiation, λ = 1.54184 Å
*a* = 12.3973 (4) Å	Cell parameters from 4194 reflections
*b* = 4.76620 (15) Å	θ = 3.6–76.0°
*c* = 31.5382 (10) Å	µ = 0.56 mm^−^^1^
β = 99.318 (3)°	*T* = 300 K
*V* = 1838.94 (10) Å^3^	Needle, clear light black
*Z* = 2	0.24 × 0.06 × 0.04 mm

### Data collection

**Table:**

XtaLAB Synergy, Dualflex, HyPix diffractometer	3568 independent reflections
Radiation source: Rotating-anodeX-raytube	2494 reflections with *I* > 2σ(*I*)
Detector resolution: 10.0000 pixels mm^-1^	*R*_int_ = 0.035
fαndωscans	θ_max_ = 76.5°, θ_min_ = 2.8°
Absorption correction: multi-scan (CrysAlisPro; Rigaku OD, 2022)	*h* = −15→15
*T*_min_ = 0.288, *T*_max_ = 1.000	*k* = −5→4
10392 measured reflections	*l* = −36→39

### Refinement

**Table:**

Refinement on *F*^2^	Primary atom site location: dual
Least-squares matrix: full	Hydrogen site location: inferred from neighbouring sites
*R*[*F*^2^ > 2σ(*F*^2^)] = 0.090	H-atom parameters constrained
*wR*(*F*^2^) = 0.255	*w* = 1/[σ^2^(*F*_o_^2^) + (0.1846*P*)^2^] where *P* = (*F*_o_^2^ + 2*F*_c_^2^)/3
*S* = 1.05	(Δ/σ)_max_ = 0.001
3568 reflections	Δρ_max_ = 0.23 e Å^−^^3^
218 parameters	Δρ_min_ = −0.34 e Å^−^^3^
0 restraints	

### Special details

**Table:**

Geometry. All esds (except the esd in the dihedral angle between two l.s. planes) are estimated using the full covariance matrix. The cell esds are taken into account individually in the estimation of esds in distances, angles and torsion angles; correlations between esds in cell parameters are only used when they are defined by crystal symmetry. An approximate (isotropic) treatment of cell esds is used for estimating esds involving l.s. planes.

### Fractional atomic coordinates and isotropic or equivalent isotropic displacement parameters (Å^2^)

**Table:**

	*x*	*y*	*z*	*U*_iso_*/*U*_eq_	
O1	0.36140 (17)	0.5877 (4)	0.44237 (5)	0.1191 (7)	
N1	0.32675 (13)	0.7409 (3)	0.50605 (5)	0.0727 (5)	
N2	−0.0384 (2)	−0.2956 (5)	0.38938 (10)	0.1224 (8)	
C7	0.51047 (16)	1.0897 (3)	0.51924 (6)	0.0707 (5)	
C4	0.34537 (16)	0.9202 (4)	0.54140 (6)	0.0744 (5)	
C3	0.23135 (17)	0.5896 (4)	0.50748 (7)	0.0832 (6)	
C5	0.39073 (17)	0.7351 (4)	0.47350 (6)	0.0788 (6)	
C6	0.43898 (16)	1.0958 (4)	0.54828 (6)	0.0715 (5)	
C15	0.45368 (17)	1.2647 (4)	0.58936 (6)	0.0786 (6)	
H15A	0.3828	1.3005	0.5975	0.094*	
H15B	0.4867	1.4440	0.5846	0.094*	
C10	0.09962 (17)	0.0370 (4)	0.41748 (8)	0.0877 (6)	
C1	0.26186 (19)	0.8770 (4)	0.56470 (8)	0.0874 (6)	
H1	0.2532	0.9685	0.5900	0.105*	
C11	0.0089 (2)	−0.1250 (5)	0.41994 (9)	0.0959 (7)	
H11	−0.0215	−0.1137	0.4450	0.115*	
C8	0.18723 (18)	0.3892 (4)	0.47647 (8)	0.0891 (7)	
C2	0.1927 (2)	0.6726 (5)	0.54376 (9)	0.0948 (7)	
H2	0.1303	0.6034	0.5530	0.114*	
C16	0.5257 (2)	1.1103 (4)	0.62572 (6)	0.0847 (6)	
H16A	0.4877	0.9427	0.6327	0.102*	
H16B	0.5922	1.0512	0.6158	0.102*	
C12	0.0069 (3)	−0.3101 (8)	0.35413 (13)	0.1385 (12)	
H12	−0.0252	−0.4274	0.3321	0.166*	
C9	0.14449 (18)	0.2220 (4)	0.45104 (8)	0.0920 (7)	
C17	0.5558 (2)	1.2804 (5)	0.66556 (7)	0.1010 (8)	
H17A	0.4892	1.3449	0.6749	0.121*	
H17B	0.5956	1.4450	0.6587	0.121*	
C18	0.6239 (2)	1.1297 (6)	0.70235 (7)	0.1040 (8)	
H18A	0.5835	0.9655	0.7090	0.125*	
H18B	0.6898	1.0636	0.6927	0.125*	
C19	0.6565 (3)	1.2890 (7)	0.74240 (9)	0.1299 (12)	
H19A	0.5905	1.3511	0.7524	0.156*	
H19B	0.6952	1.4556	0.7356	0.156*	
C14	0.1440 (3)	0.0156 (9)	0.38057 (11)	0.1317 (11)	
H14	0.2051	0.1210	0.3771	0.158*	
C13	0.0965 (3)	−0.1660 (10)	0.34829 (13)	0.1589 (15)	
H13	0.1261	−0.1870	0.3232	0.191*	
C20	0.7258 (4)	1.1427 (10)	0.77840 (10)	0.1596 (17)	
H20A	0.6901	0.9677	0.7835	0.192*	
H20B	0.7945	1.0954	0.7692	0.192*	
C21	0.7502 (5)	1.2915 (11)	0.81890 (12)	0.188 (2)	
H21A	0.7859	1.4656	0.8146	0.282*	
H21B	0.7974	1.1788	0.8393	0.282*	
H21C	0.6835	1.3287	0.8297	0.282*	

### Atomic displacement parameters (Å^2^)

**Table:**

	*U*^11^	*U*^22^	*U*^33^	*U*^12^	*U*^13^	*U*^23^
O1	0.1286 (15)	0.1399 (14)	0.0870 (10)	−0.0598 (12)	0.0124 (10)	−0.0351 (10)
N1	0.0735 (9)	0.0680 (8)	0.0707 (9)	−0.0054 (6)	−0.0064 (7)	0.0048 (6)
N2	0.1047 (16)	0.1198 (16)	0.134 (2)	−0.0282 (13)	−0.0080 (15)	−0.0193 (14)
C7	0.0774 (11)	0.0665 (9)	0.0618 (9)	−0.0030 (8)	−0.0083 (8)	0.0020 (7)
C4	0.0786 (11)	0.0704 (9)	0.0691 (10)	0.0029 (8)	−0.0034 (9)	0.0059 (7)
C3	0.0769 (12)	0.0730 (10)	0.0930 (14)	−0.0106 (9)	−0.0064 (10)	0.0129 (9)
C5	0.0828 (12)	0.0779 (11)	0.0694 (11)	−0.0107 (9)	−0.0067 (9)	−0.0006 (8)
C6	0.0772 (11)	0.0672 (9)	0.0639 (9)	0.0028 (8)	−0.0071 (8)	0.0044 (7)
C15	0.0868 (13)	0.0763 (10)	0.0676 (11)	0.0057 (9)	−0.0032 (9)	−0.0035 (8)
C10	0.0705 (11)	0.0816 (11)	0.1037 (15)	−0.0044 (9)	−0.0073 (11)	0.0043 (11)
C1	0.0882 (14)	0.0868 (12)	0.0859 (12)	0.0009 (10)	0.0100 (11)	0.0094 (10)
C11	0.0850 (14)	0.0896 (13)	0.1082 (16)	−0.0149 (11)	0.0008 (12)	−0.0049 (12)
C8	0.0763 (12)	0.0753 (11)	0.1081 (16)	−0.0108 (9)	−0.0082 (11)	0.0152 (10)
C2	0.0845 (14)	0.0918 (13)	0.1073 (17)	−0.0084 (11)	0.0131 (12)	0.0155 (12)
C16	0.0983 (14)	0.0830 (12)	0.0673 (11)	0.0077 (10)	−0.0031 (10)	0.0001 (8)
C12	0.126 (3)	0.152 (3)	0.129 (3)	−0.018 (2)	−0.004 (2)	−0.036 (2)
C9	0.0744 (12)	0.0784 (11)	0.1149 (17)	−0.0124 (9)	−0.0097 (12)	0.0100 (11)
C17	0.1199 (19)	0.1020 (15)	0.0712 (13)	0.0131 (13)	−0.0143 (12)	−0.0085 (10)
C18	0.1181 (19)	0.1188 (17)	0.0680 (12)	0.0100 (14)	−0.0066 (12)	0.0023 (11)
C19	0.151 (3)	0.141 (2)	0.0823 (16)	0.0299 (19)	−0.0262 (17)	−0.0199 (14)
C14	0.0939 (18)	0.171 (3)	0.132 (2)	−0.0288 (19)	0.0242 (17)	−0.007 (2)
C13	0.135 (3)	0.218 (4)	0.127 (3)	−0.023 (3)	0.032 (2)	−0.048 (3)
C20	0.186 (4)	0.195 (4)	0.0822 (17)	0.057 (3)	−0.023 (2)	−0.0110 (19)
C21	0.225 (5)	0.216 (4)	0.101 (2)	0.065 (4)	−0.044 (3)	−0.030 (2)

### Geometric parameters (Å, º)

**Table:**

O1—C5	1.214 (2)	C2—H2	0.9300
N1—C3	1.392 (3)	C16—C17	1.491 (3)
N1—C4	1.394 (2)	C16—H16A	0.9700
N1—C5	1.396 (3)	C16—H16B	0.9700
N2—C11	1.323 (3)	C12—C13	1.343 (6)
N2—C12	1.327 (5)	C12—H12	0.9300
C7—C6	1.374 (3)	C17—C18	1.502 (3)
C7—C5^i^	1.470 (3)	C17—H17A	0.9700
C7—C7^i^	1.473 (3)	C17—H17B	0.9700
C4—C1	1.378 (3)	C18—C19	1.473 (3)
C4—C6	1.419 (3)	C18—H18A	0.9700
C3—C2	1.368 (4)	C18—H18B	0.9700
C3—C8	1.413 (3)	C19—C20	1.483 (4)
C5—C7^i^	1.470 (3)	C19—H19A	0.9700
C6—C15	1.511 (3)	C19—H19B	0.9700
C15—C16	1.524 (3)	C14—C13	1.392 (5)
C15—H15A	0.9700	C14—H14	0.9300
C15—H15B	0.9700	C13—H13	0.9300
C10—C14	1.370 (4)	C20—C21	1.450 (5)
C10—C11	1.377 (3)	C20—H20A	0.9700
C10—C9	1.420 (3)	C20—H20B	0.9700
C1—C2	1.392 (3)	C21—H21A	0.9600
C1—H1	0.9300	C21—H21B	0.9600
C11—H11	0.9300	C21—H21C	0.9600
C8—C9	1.192 (3)		
			
C3—N1—C4	108.86 (18)	C15—C16—H16B	108.7
C3—N1—C5	126.90 (16)	H16A—C16—H16B	107.6
C4—N1—C5	124.08 (16)	N2—C12—C13	124.1 (3)
C11—N2—C12	116.4 (3)	N2—C12—H12	118.0
C6—C7—C5^i^	119.69 (17)	C13—C12—H12	118.0
C6—C7—C7^i^	121.0 (2)	C8—C9—C10	173.9 (3)
C5^i^—C7—C7^i^	119.3 (2)	C16—C17—C18	115.0 (2)
C1—C4—N1	107.06 (17)	C16—C17—H17A	108.5
C1—C4—C6	132.29 (19)	C18—C17—H17A	108.5
N1—C4—C6	120.62 (19)	C16—C17—H17B	108.5
C2—C3—N1	106.95 (18)	C18—C17—H17B	108.5
C2—C3—C8	128.7 (2)	H17A—C17—H17B	107.5
N1—C3—C8	124.3 (2)	C19—C18—C17	117.2 (2)
O1—C5—N1	118.36 (19)	C19—C18—H18A	108.0
O1—C5—C7^i^	126.0 (2)	C17—C18—H18A	108.0
N1—C5—C7^i^	115.64 (16)	C19—C18—H18B	108.0
C7—C6—C4	119.04 (17)	C17—C18—H18B	108.0
C7—C6—C15	125.57 (18)	H18A—C18—H18B	107.2
C4—C6—C15	115.30 (19)	C18—C19—C20	117.3 (3)
C6—C15—C16	111.27 (15)	C18—C19—H19A	108.0
C6—C15—H15A	109.4	C20—C19—H19A	108.0
C16—C15—H15A	109.4	C18—C19—H19B	108.0
C6—C15—H15B	109.4	C20—C19—H19B	108.0
C16—C15—H15B	109.4	H19A—C19—H19B	107.2
H15A—C15—H15B	108.0	C10—C14—C13	119.1 (3)
C14—C10—C11	116.8 (2)	C10—C14—H14	120.4
C14—C10—C9	121.1 (2)	C13—C14—H14	120.4
C11—C10—C9	122.1 (3)	C12—C13—C14	118.6 (4)
C4—C1—C2	108.0 (2)	C12—C13—H13	120.7
C4—C1—H1	126.0	C14—C13—H13	120.7
C2—C1—H1	126.0	C21—C20—C19	117.2 (3)
N2—C11—C10	125.0 (3)	C21—C20—H20A	108.0
N2—C11—H11	117.5	C19—C20—H20A	108.0
C10—C11—H11	117.5	C21—C20—H20B	108.0
C9—C8—C3	176.3 (3)	C19—C20—H20B	108.0
C3—C2—C1	109.1 (2)	H20A—C20—H20B	107.2
C3—C2—H2	125.5	C20—C21—H21A	109.5
C1—C2—H2	125.5	C20—C21—H21B	109.5
C17—C16—C15	114.06 (17)	H21A—C21—H21B	109.5
C17—C16—H16A	108.7	C20—C21—H21C	109.5
C15—C16—H16A	108.7	H21A—C21—H21C	109.5
C17—C16—H16B	108.7	H21B—C21—H21C	109.5
			
C3—N1—C4—C1	0.5 (2)	C7—C6—C15—C16	−83.8 (2)
C5—N1—C4—C1	176.21 (16)	C4—C6—C15—C16	92.7 (2)
C3—N1—C4—C6	178.77 (15)	N1—C4—C1—C2	0.1 (2)
C5—N1—C4—C6	−5.5 (3)	C6—C4—C1—C2	−177.94 (19)
C4—N1—C3—C2	−0.9 (2)	C12—N2—C11—C10	0.6 (4)
C5—N1—C3—C2	−176.42 (18)	C14—C10—C11—N2	−0.7 (4)
C4—N1—C3—C8	179.55 (17)	C9—C10—C11—N2	178.1 (2)
C5—N1—C3—C8	4.0 (3)	N1—C3—C2—C1	0.9 (2)
C3—N1—C5—O1	1.3 (3)	C8—C3—C2—C1	−179.55 (19)
C4—N1—C5—O1	−173.68 (18)	C4—C1—C2—C3	−0.6 (3)
C3—N1—C5—C7^i^	−178.28 (15)	C6—C15—C16—C17	172.3 (2)
C4—N1—C5—C7^i^	6.8 (3)	C11—N2—C12—C13	0.5 (6)
C5^i^—C7—C6—C4	−178.44 (15)	C15—C16—C17—C18	178.2 (2)
C7^i^—C7—C6—C4	1.8 (3)	C16—C17—C18—C19	179.6 (3)
C5^i^—C7—C6—C15	−2.1 (3)	C17—C18—C19—C20	−178.6 (3)
C7^i^—C7—C6—C15	178.13 (18)	C11—C10—C14—C13	−0.3 (5)
C1—C4—C6—C7	178.65 (19)	C9—C10—C14—C13	−179.2 (3)
N1—C4—C6—C7	0.9 (2)	N2—C12—C13—C14	−1.5 (7)
C1—C4—C6—C15	1.9 (3)	C10—C14—C13—C12	1.4 (6)
N1—C4—C6—C15	−175.86 (14)	C18—C19—C20—C21	−175.0 (4)

Symmetry code: (i) −*x*+1, −*y*+2, −*z*+1.
