# Supplementary figures and images for: 6,6′-Diheptyl-3,3′-bis­[(pyridin-3-yl)ethyn­yl]-5H,5′H-di­pyrrolo­[1,2-b:1′,2′-g][2,6]naphthyridine-5,5′-dione
Source: IUCrdata. 2023 Jun 9;8(Pt 6):x230513. doi: 10.1107/S2414314623005138 (PMC10626619; doi:10.1107/S2414314623005138)

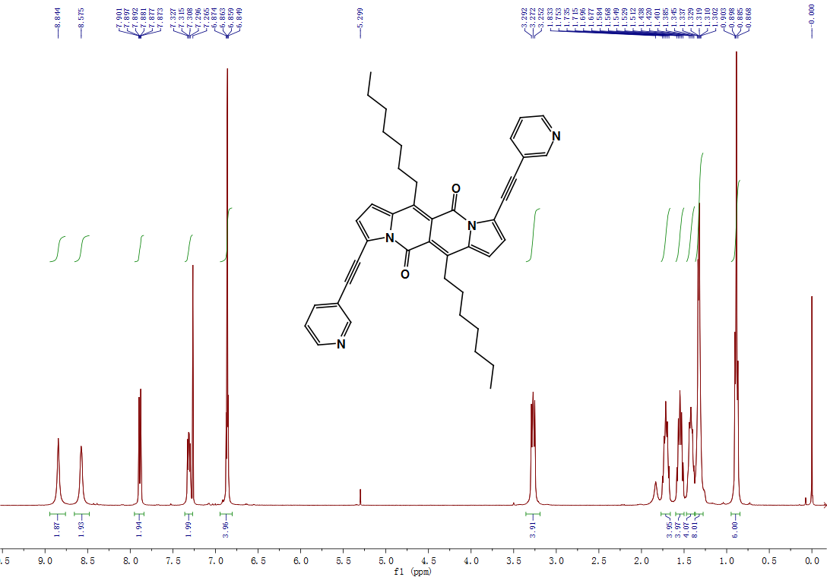

Supplement: Supplementary file 3 [file x-08-x230513-Isup4.tif]
